# AccessPhysiotherapy

**DOI:** 10.5195/jmla.2017.193

**Published:** 2017-07-01

**Authors:** Carol Lewis Watwood

## OVERVIEW

Launched in 2010, AccessPhysiotherapy is a multimedia online educational resource targeted to physical therapy students, educators, and practitioners [1]. It is especially suitable for students who are enrolled in doctoral-level physiotherapy programs or preparing for the National Physical Therapy Examination. Part of the Access suite of online educational products from McGraw-Hill Medical, AccessPhysiotherapy also includes the more widely known AccessMedicine, JAMAevidence, and many other specialty offerings. AccessPhysiotherapy includes books, quick reference materials, drug information, a video library, images, examination reviews, an interactive online dissection tool, neuroscience lectures, and case studies. A custom curriculum option allows students and faculty to sign in and manage content tailored to a specific program.

The Advisory Board for AccessPhysiotherapy consists of Annie Burke-Doe of the University of St. Augustine for Health Science in San Diego, California; Eric Shamus, associate professor in the Florida Gulf Coast University Doctor of Physical Therapy program; and Mark Dutton, a physical therapist with Allegheny General Hospital, West Penn Health System, in Pittsburgh, Pennsylvania. Besides their advisory role, the editorial board members have contributed substantial amounts of content to AccessPhysiotherapy and are all authors of well-known physical therapy texts.

AccessPhysiotherapy was easy to set up for in-network and remote institutional use via Internet protocol (IP) authentication at the author’s university library. In addition to IP authentication, access via EZProxy, Athens authentication, referral uniform resource locator (URL), and/or username and password is available. The site works well on Windows, MacOS, and Android operating systems and with a variety of browsers. The display automatically resizes to fit mobile device screens [2]. Users can create optional individual MyAccess accounts to access self-assessment, save materials to “Favorites,” review recently viewed content, and download images into PowerPoint slides. Off-site users who have created a MyAccess account while logged in to their institutions can sign in later with just their MyAccess login.

Links to printable color flyers, brochures, and video tutorials are provided by McGraw-Hill at the time of purchase. COUNTER-compliant statistical reports are available, clearly labeled, and easy to read.

**Figure 1:**
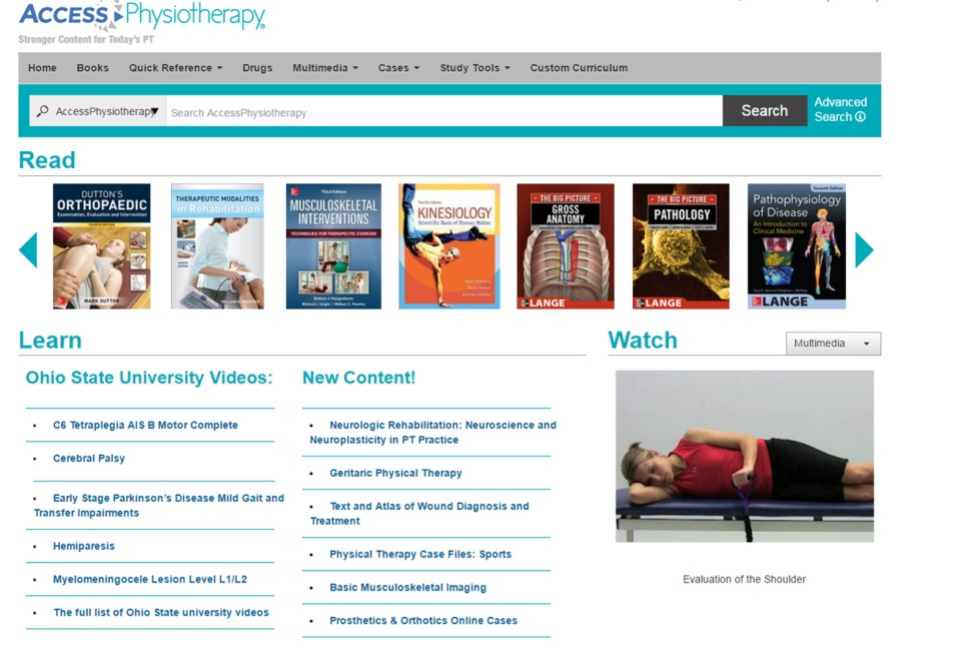
AccessPhysiotherapy home page

AccessPhysiotherapy features a clean, colorful home page with a row of tabs grouping content into broad categories at the top of the page: Home, Books, Quick Reference, Drugs, Multimedia, Cases, Study Tools, and Custom Curriculum (Figure 1). Also from the top-level page, users can browse a gallery of available books, search the site, or access guides to new and featured content and a site directory. Students at our institution have commented that the AccessPhysiotherapy website is easy to navigate.

The basic/advanced search box appearing near the top of the screen allows Boolean and phrase searching. Clicking an arrow displays a menu option that allows federated searching of all Access sites. Results are displayed in context by resource title and can be filtered by type of resource, book title, or topic. The search can be expanded to other AccessMedicine and other McGraw-Hill Medical sites. Interestingly, preliminary statistics at the author’s institution show little use of the search box. Overwhelmingly, users access individual book titles and other resources directly.

## ONTENT

The Books tab displays available books by title and subject. A core feature of AccessPhysiotherapy is its searchable online collection of McGraw-Hill physical therapy text-books and compilations of case studies. Books with related content in orthopedic examination, pathology, biostatistics, pathophysiology, imaging, and other subjects pertinent to physical therapy are also included. The selection of books has been expanded and currently includes thirty-nine McGraw-Hill titles. Faculty may use the collection to supplement or replace textbooks used in a physical therapy program to guarantee easy, portable online access to course textbooks for all students. MARC 21 records are provided for books included in AccessPhysiotherapy so that records can be included in the library catalog. OpenURL links to PubMed records have been added to many references. A book list can be downloaded in Excel format.

When a textbook is selected, the contents are displayed in expandable sections. Textbook chapters may be printed, emailed, or searched by keyword. Full-text textbook content is not downloadable. American Medical Association (AMA)– and Modern Language Association (MLA)–style citations can be displayed or downloaded. Images and tables that have been approved for educational use may be bookmarked or downloaded to PowerPoint slides. The screen display is clean and easy to navigate. Clickable links to related content and online updates are displayed on the right-hand side of the page.

A useful Quick Answers section, available under Quick Reference, provides detailed point-of-care information on conditions of interest to physical therapists. It includes items such as the International Classification of Diseases, Ninth Revision (ICD-9-CM), and ICD-10-CM codes; links to material on the American Physical Therapy Association website; and information on diagnosis, therapy, prognosis, tests, goals, and references with live links to pertinent materials in AccessPhysiotherapy. The “Outcome Measures Toolbox” links to a variety of scales and measures used to assess physical therapy outcomes, with live links to other AccessPhysiotherapy resources. Calculators allow users to compute body mass index (BMI) and other common measurements.

A Multimedia section includes Anatomy and Physiology Revealed, a sophisticated interactive cadaver tool from the University of Toledo. Students can peel away layers of realistic-looking virtual cadaver organs to study human anatomy. This feature also includes animations and videos illustrating body functions and disease processes. Other multimedia content, which is updated periodically, includes videos, interactive modules, and lectures. Multimedia content comes from McGraw-Hill products, university physical therapy departments, and other sources.

The Study Tools feature can be used to generate custom multiple-choice practice quizzes for the National Physical Therapy Examination. Tests and quizzes can also be generated and scored for classroom use or self-assessment. Students can sign in through MyAccess and review their progress through a feature called My Review Questions. This targeted test review function is a key value-added feature of AccessPhysiotherapy. The Custom Curriculum feature, also accessible via MyAccess, can be used for assignments and course management. If another course management system is already in use, this feature may be redundant.

The F.A. Davis PT Collection provides additional text-books, cases, and videos. This collection currently includes twenty-nine additional titles and is available at an additional cost. Many Davis titles are widely available through other vendors, so libraries may wish to comparison shop.

## RECOMMENDATIONS

AccessPhysiotherapy is an attractively packaged niche product with little direct competition. The most valuable component is the full-text McGraw-Hill physiotherapy e-book collection, which is not widely available else-where. Test preparation is another valuable function of AccessPhysiotherapy that has enjoyed good usage by our students. AccessPhysiotherapy’s online anatomy tool and other multi-media content also add value. Furthermore, for institutions subscribing to AccessMedicine and other McGraw-Hill products, the ability to search multiple products simultaneously with AccessPhysiotherapy is an advantage.

Current institutional pricing for AccessPhysiotherapy is a good value for the amount of information that it provides. The major drawback is that e-book content is limited to McGraw-Hill and Davis products and, therefore, does not provide comprehensive coverage. Indeed, only one of the 2016 Doody’s Core Titles recommended in the “Physical Therapy” section appears in AccessPhysiotherapy (four, if the Davis PT add-on is included) [3]. The e-book collections of Ovid, ClinicalKey, EBSCO e-books, Stat!Ref, R2 Digital Library, and others in addition to or instead of AccessPhysiotherapy provide access to other core physiotherapy titles but require users to learn multiple interfaces. Users who desire an e-book collection only and do not need the value-added features of AccessPhysiotherapy might compare pricing of the McGraw-Hill Education eBook Library.

Given this strong caveat, AccessPhysiotherapy merits a thirty-day trial for institutions with a doctoral-level physical therapy program or other academic and clinical physical therapy programs with a need for one-stop-shop physical therapy information. If faculty like and use a good portion of the material, the easy-to-read online textbook display, study tools, and McGraw-Hill e-book collection that AccessPhysiotherapy comprises are worth the relatively moderate subscription price. Its compatibility and inter-face with AccessMedicine and other McGraw-Hill products should increase its chances of surviving in the increasingly competitive e-book market. In summary, AccessPhysiotherapy is a well-designed, easy-to-use product based on McGraw-Hill physical therapy and related textbook content, with some audiovisual and other content add-ins. It offers an appealing, portable platform for physiotherapy students to study textbooks, view videos, and prepare for exams.
